# A Daily Snack Containing Leafy Green Vegetables, Fruit, and Milk before and during Pregnancy Prevents Gestational Diabetes in a Randomized, Controlled Trial in Mumbai, India^1^,^2^,^3^,^4^

**DOI:** 10.3945/jn.115.223461

**Published:** 2016-06-08

**Authors:** Sirazul A Sahariah, Ramesh D Potdar, Meera Gandhi, Sarah H Kehoe, Nick Brown, Harshad Sane, Patsy J Coakley, Ella Marley-Zagar, Harsha Chopra, Devi Shivshankaran, Vanessa A Cox, Alan A Jackson, Barrie M Margetts, Caroline HD Fall

**Affiliations:** 5 Centre for the Study of Social Change, Mumbai, India; and; 6 MRC Lifecourse Epidemiology Unit,; 7 NIHR Southampton Biomedical Research Centre, and; 8 Public Health Nutrition, Faculty of Medicine, University of Southampton, Southampton, United Kingdom

**Keywords:** randomized controlled trial, food-based supplement, leafy green vegetables, fruit, milk, micronutrients, pregnancy, gestational diabetes, India

## Abstract

**Background:** Prospective observational studies suggest that maternal diets rich in leafy green vegetables and fruit may help prevent gestational diabetes mellitus (GDM).

**Objective:** Our objective was to test whether increasing women’s dietary intake of leafy green vegetables, fruit, and milk before conception and throughout pregnancy reduced their risk of GDM.

**Methods:** Project SARAS (“excellent”) (2006–2012) was a nonblinded, individually randomized, controlled trial in women living in slums in the city of Mumbai, India. The interventions included a daily snack made from leafy green vegetables, fruit, and milk for the treatment group or low-micronutrient vegetables (e.g., potato and onion) for the control group, in addition to the usual diet. Results for the primary outcome, birth weight, have been reported. Women were invited to take an oral-glucose-tolerance test (OGTT) at 28–32 wk gestation to screen for GDM (WHO 1999 criteria). The prevalence of GDM was compared between the intervention and control groups, and Kernel density analysis was used to compare distributions of 120-min plasma glucose concentrations between groups.

**Results:** Of 6513 women randomly assigned, 2291 became pregnant; of these, 2028 reached a gestation of 28 wk, 1008 (50%) attended for an OGTT, and 100 (9.9%) had GDM. In an intention-to-treat analysis, the prevalence of GDM was reduced in the treatment group (7.3% compared with 12.4% in controls; OR: 0.56; 95% CI: 0.36, 0.86; *P* = 0.008). The reduction in GDM remained significant after adjusting for prepregnancy adiposity and fat or weight gain during pregnancy. Kernel density analysis showed that this was explained by the fact that fewer women in the treatment group had a 2-h glucose concentration in the range 7.5–10.0 mmol/L.

**Conclusions:** In low-income settings, in which women have a low intake of micronutrient-rich foods, improving dietary micronutrient quality by increasing intake of leafy green vegetables, fruit, and/or milk may have an important protective effect against the development of GDM. This trial was registered at www.controlled-trials.com as ISRCTN62811278.

## Introduction

Gestational diabetes mellitus (GDM)^9^ is a common disorder of pregnancy associated with increased risks for the mother [obstructed labor and later type 2 diabetes mellitus (T2DM)] and baby (congenital malformations, macrosomia, and neonatal hypoglycemia). Offspring of mothers with GDM have an increased risk of developing adult obesity and T2DM (1). Risk factors for GDM are similar to those for T2DM (older age and greater adiposity) and the prevalence is rising everywhere (2). The Hyperglycemia and Adverse Pregnancy Outcomes study showed that the complications of GDM increase linearly across the range of plasma glucose values, leading to debate about the best clinical criteria for diagnosing GDM (3, 4). Although improved management of GDM reduces obstetric complications (5), treatment can be onerous, and it is unknown whether long-term complications in the offspring are reduced (6). Strategies to prevent the disease are therefore needed.

Observational studies suggest that maternal diet may influence GDM risk (7–9). A lower intake of saturated fat, red or processed meat, refined grains, and sweets, and a higher intake of fiber, fruit, vegetables, poultry, and fish, before or during pregnancy, is associated with a lower prevalence (7, 8). Higher plasma vitamin B-12, C, and D concentrations have also been associated with lower risk (7, 9). There is similar evidence for T2DM (10, 11), and prospective studies have shown that a higher intake of dairy products (12) and leafy green vegetables (13, 14) predict lower risk. Observational studies are subject to confounding, and these findings need to be tested in randomized intervention studies. However, most dietary trials to prevent GDM have focused on reduction in weight gain during pregnancy, with little impact (15, 16).

South Asians are at high risk of GDM, and studies in India have recorded prevalence rates of 6–17% in urban populations (17). Project SARAS (“excellent”) was a randomized, controlled trial in India in which women’s diets were supplemented with a daily snack made from leafy green vegetables, fruit, and milk, preconceptionally and throughout pregnancy. The primary objective was to increase birth weight. In the intention-to-treat analysis, there was no overall increase in birth weight; however, there was an interaction (*P* < 0.001) with maternal prepregnancy BMI such that birth weight increased by 63 g (95% CI: 11, 115 g) in the treatment group compared with controls in mothers of normal or high prepregnancy BMI (in kg/m^2^; >18.5) (18). Women were offered an oral-glucose-tolerance test (OGTT) at 28–32 wk gestation because, although not a primary outcome, GDM status was an important covariate for the interpretation of supplementation effects on birth weight. The OGTT data enabled us to test whether the intervention benefited maternal metabolism as assessed by glucose tolerance.

## Methods

### 

#### Setting and participants.

The trial took place from 2006 to 2012 in slums in the city of Mumbai, India (18) (see **Supplemental Trial Protocol**). Women were eligible if they were aged <40 y, married, not pregnant, not sterilized, planning to have more children, and intending to deliver in Mumbai.

#### Intervention.

The intervention was a daily snack resembling local street foods such as samosas and fritters, prepared fresh each day and fried in sunflower oil. Treatment snacks contained leafy green vegetables in fresh (∼30 g) or dried (∼7.5 g) form, full-fat milk powder (12–16 g), and dried fruits (4–60 g) (**Supplemental Tables 1** and **2**). Control snacks were made from low-micronutrient vegetables such as potato and onion. To avoid monotony, we created multiple recipes from these foods (**Supplemental Table 3**). On average, treatment snacks contained 10–23% of the WHO/FAO recommended Reference Nutrient Intake for β-carotene, riboflavin, folate, vitamin B-12, calcium, and iron; they contained 0.69 MJ energy and 6.4 g protein, compared with 0.37 MJ and 2.4 g in control snacks (Supplemental Table 2) (19, 20).

#### Recruitment, baseline investigations, and randomization.

Women were screened for eligibility, and individual written informed consent was obtained. We recorded education, occupation, and socioeconomic status with the use of the Standard of Living Index (21). Tobacco use was recorded. Diet was assessed with the use of an FFQ, with the reference period the preceding week (22). Weight, height, and triceps and subscapular skinfolds were measured with the use of standardized techniques. Women were individually randomly assigned and stratified by age and BMI (3 groups for each) (18).

#### Blinding.

Full blinding is impossible in a food-based trial. Although treatment and control snacks were outwardly similar, their contents looked different. To obscure allocation, we created 2 treatment and 2 control groups, each with its own recipes, which were merged for the analysis. Four different snacks therefore were produced daily. Staff who measured outcomes were blind to the women’s allocation group.

#### Supplementation.

Snacks were produced daily except on holidays, packaged in color-coded bags, and transported to 61 supplementation centers. Women were asked to maintain their usual diet, and snacks were available from 1500 to 1800, to interfere least with meals. Women were offered 1 snack/d, and consumption was observed and recorded. Center staff recorded the women’s serial last menstrual period (LMP) dates. Compliance was defined as a mean of ≥3 snacks/wk from 90 d before the LMP date until delivery.

#### Pregnancies and OGTTs.

Women who became pregnant were prescribed iron (100 mg) and folic acid (500 μg) in accordance with national guidelines. At a gestation of 9–13 wk, blood was collected to measure hemoglobin and plasma vitamin B-12 and folate. Plasma cobalamin (B-12) and folate were measured with the use of microbiological assays (23–26). At a gestation of 28–32 wk, women were offered an OGTT. Venous blood was collected after an overnight fast and 120 min after 75 g anhydrous glucose orally in water. Glucose samples were collected into fluoride tubes and analyzed by autoanalyzer (ERBA EM200; Transasia) in a single Mumbai laboratory within 6 h of venesection. Fasting insulin samples were placed on ice and centrifuged within 3 h at 450–700 × *g* for 20 min at room temperature; plasma aliquots were stored at −80°C until analysis with the use of ELISA kits (Mercodia Ultrasensitive Insulin kits; Mercodia AB) with inter- and intra-assay CVs <7%. GDM was diagnosed based on WHO 1999 criteria [fasting glucose ≥7.0 mmol/L (126 mg/dL) and/or 120-min glucose ≥7.8 mmol/L (140 mg/dL)] (27). Women were referred to their own obstetricians for further GDM management.

#### Deliveries.

Trained research nurses measured birth weight. Gestational age was calculated from the LMP date unless different by >14 d from that estimated by an ultrasound scan (9%) at <20 wk, when the latter was used (28).

#### Outcomes.

Outcomes for this analysis were GDM, fasting and 120-min glucose concentrations, and fasting insulin concentration. During the trial we used the 1999 WHO definition of GDM (27). In 2013, the definition changed (29) to match the International Association of Diabetes and Pregnancy Study Groups Consensus Panel recommendations (30) as follows: fasting glucose concentration of 5.1–6.9 mmol/L (92–125 mg/dL) and/or a 120-min glucose concentration of 8.5–11.0 mmol/L (153–199 mg/dL) and a category of “diabetes in pregnancy” was introduced [fasting glucose concentration ≥7.0 (126 mg/dL) and 120-min glucose concentration ≥11.1 mmol/L (200 mg/dL)]. We report results for both 1999 (27) and 2013 (29) criteria.

#### Ethical approval and governance.

The trial (ISRCTN62811278) was approved by the ethics committees of BYL Nair and TN Medical College, Grant Medical College, and Sir JJ Group of Hospitals, Mumbai, and Southampton and SW local research ethics committees. An independent data monitoring committee reviewed data on compliance, completeness of follow-up, pregnancy outcomes, and adverse events every 6 mo for the first 3 y of the trial and then every year.

#### Statistical methods.

We compared baseline measurements between women who did and did not have an OGTT, and between allocation groups. We compared outcomes between allocation groups in all women who were randomly assigned, became pregnant and had an OGTT (intention-to-treat analysis), and limited to women who started supplementation >90 d before their LMP date (per-protocol analysis) (**Supplemental Figure 1**). We tested for interactions between allocation group and maternal age, BMI, height, and parity. Small-for-gestational-age and large-for-gestational-age births were defined in accordance with Oken et al. (31) and also “within-cohort” as <10th and >90th percentile based on singleton live births without major congenital abnormalities. Preterm birth was defined as gestation <37 wk. A *t* test, Mann-Whitney *U* test, or chi-square or Fisher’s exact test was used to compare groups for normally distributed continuous, nonparametric, and categorical variables, respectively; further comparisons of glucose concentrations between groups were made with the use of Kernel density estimates. Main results are reported unadjusted; we then used multiple regression to assess intervention effects on GDM while adjusting for maternal age, adiposity (subscapular skinfold thickness and/or weight at recruitment and subscapular skinfold gain and/or weight gain from recruitment to 28 wk gestation), height, parity, socioeconomic status, and compliance. Analysis was performed with the use of STATA version 13.0.

## Results

A total of 6513 nonpregnant women participated in the trial, of whom 2291 became pregnant (Supplemental Figure 1). Of these, 241 had either an abortion or termination of pregnancy before 28 wk gestation, and we lost contact with 22 women. Of the remaining 2028, 1008 (50%) attended for an OGTT at a median (IQR) gestation of 29.7 (29.3, 30.4) wk. Women who did not have an OGTT were younger and of lower socioeconomic status and parity than women who attended (**Table 1**). However, there were only small differences in characteristics between allocation groups; in women who had an OGTT, those in the treatment group had lower baseline weight, BMI, and subscapular skinfold thickness than did controls; they also had thinner skinfolds at visit 1, but greater pregnancy weight gain (Table 1).

**TABLE 1:** Comparison of baseline characteristics between women who did and did not attend for an OGTT and between treatment and control groups^1^

	Baseline comparison					Group comparison			
	Attended for OGTT (*n* = 1008)		Did not attend for OGTT (*n* = 1020)			Attended for OGTT		Did not attend for OGTT	
	*n*	Value	*n*	Value	*P*	Treatment (*n* = 492)	Control (*n* = 516)	Treatment (*n* = 497)	Control (*n* = 523)
At recruitment (prepregnancy)									
Age, y	1008	24.0 (21.0, 27.0)	1020	23.0 (21.0, 26.0)	<0.001	24.0 (21.0, 27.0)	25.0 (22.0, 27.0)	23.0 (21.0, 26.0)	23.0 (21.0, 26.0)
Weight, kg	1008	45.6 (40.0, 51.9)	1019	45.4 (40.3, 51.1)	0.78	45.1 (39.3, 51.3)*	46.1 (40.8, 52.7)	45.5 (40.4, 51.7)	45.4 (40.3, 50.8)
Height, cm	1008	151.3 ± 5.4	1019	151.4 ± 5.5	0.59	151.3 ± 5.6	151.3 + 5.2	151.5 ± 5.5	151.4 ± 5.5
BMI, kg/m^2^	1008	19.8 (17.8, 22.6)	1018	19.7 (17.9, 22.3)	0.71	19.6 (17.7, 22.3)*	20.1 (17.9, 22.8)	19.8 (17.7, 22.6)	19.6 (17.9, 22.0)
Subscapular skinfold, mm	1008	21.3 (15.2, 29.1)	1020	21.0 (15.3, 27.6)	0.30	20.7 (14.4, 27.6)*	21.6 (16.2, 30.2)	21.4 (15.4, 28.4)	20.4 (15.3, 27.2)
Parity	1008		1020		0.01				
0		304 (30.2)		364 (35.7)		155 (31.5)	149 (28.9)	197 (39.6)	167 (31.9)
1		513 (50.9)		451 (44.2)		253 (51.4)	260 (50.4)	208 (41.9)	243 (46.5)
>1		191 (18.9)		205 (20.1)		84 (17.1)	107 (20.7)	92 (18.5)	113 (21.6)
Tobacco user	1008	85 (8.4)	1020	93 (9.1)	0.59	45 (9.1)	40 (7.8)	40 (8.0)	53 (10.1)
Standard of Living Index	978	25.0 (21.0, 30.0)	990	25.0 (21.0, 29.0)	0.004	26.0 (21.0, 30.0)	25.0 (21.0, 29.0)	25.0 (21.0, 29.0)	25.0 (20.0, 29.0)
Religion	1008		1020		0.22				
Hindu		732 (72.6)		707 (69.3)		355 (72.2)	377 (73.1)	353 (71.0)	354 (67.7)
Muslim		241 (23.9)		278 (27.3)		117 (23.8)	124 (24.0)	131 (26.4)	147 (28.1)
Other		35 (3.5)		35 (3.4)		20 (4.1)	15 (2.9)	13 (2.6)	22 (4.2)
Education	1006		1020		0.07				
Primary or less		84 (8.3)		116 (11.4)		45 (9.2)	39 (7.6)	63 (12.7)	53 (10.1)
Secondary		867 (86.2)		853 (83.6)		420 (85.5)	447 (86.8)	409 (82.3)	444 (84.9)
Graduate		55 (5.5)		51 (5.0)		26 (5.3)	29 (5.6)	25 (5.0)	26 (5.0)
Occupation	1008		1020		0.001				
Semiskilled/unskilled		194 (19.2)		135 (13.2)		95 (19.3)	99 (19.2)	62 (12.5)	73 (14.0)
Skilled/self-employed		34 (3.4)		28 (2.7)		13 (2.6)	21 (4.1)	14 (2.8)	14 (2.7)
Professional		23 (2.3)		16 (1.6)		12 (2.4)	11 (2.1)	6 (1.2)	10 (1.9)
Not working/other		757 (75.1)		841 (82.5)		372 (75.6)	385 (74.6)	415 (83.5)	426 (81.5)
First language	1005		1019		<0.001				
Marathi		588 (58.5)		501 (49.2)		290 (58.9)	298 (58.1)	245 (49.4)	256 (48.9)
Hindi		324 (32.2)		412 (40.4)		158 (32.1)	166 (32.4)	196 (39.5)	216 (41.3)
Other		93 (9.3)		106 (10.4)		44 (8.9)	49 (9.6)	55 (11.1)	51 (9.8)
Dietary intake, frequency/wk									
Milk					0.18				
<1		475 (47.1)		514 (50.4)		241 (49.0)	234 (45.3)	249 (50.1)	265 (50.7)
1–6		396 (39.3)		360 (35.3)		191 (38.8)	205 (39.7)	169 (34.0)	191 (36.5)
≥7		137 (13.6)		146 (14.3)		60 (12.2)	77 (14.9)	79 (15.9)	67 (12.8)
Leafy green vegetables					0.76				
<1		236 (23.4)		252 (24.7)		121 (24.6)	115 (22.3)	124 (24.9)	128 (24.5)
1–6		746 (74.0)		740 (72.5)		357 (72.6)	389 (75.4)	358 (72.0)	382 (73.0)
≥7		26 (2.6)		28 (2.7)		14 (2.8)	12 (2.3)	15 (3.0)	13 (2.5)
Fruit					0.64				
<1		161 (16.0)		176 (17.3)		73 (14.8)	88 (17.1)	84 (16.9)	92 (17.6)
1–6		692 (68.7)		681 (66.8)		335 (68.1)	357 (69.2)	333 (67.0)	348 (66.5)
≥7		155 (15.4)		163 (16.0)		84 (17.1)	71 (13.8)	80 (16.1)	83 (15.9)
At visit 1^2^									
Weight, kg	861	46.9 (41.3, 53.5)	625	46.2 (40.7, 52.3)	0.21	46.4 (40.1, 53.1)	47.5 (41.9, 53.8)	46.2 (40.9, 53.0)	46.2 (40.6, 51.5)
Triceps skinfold, mm	898	13.9 (9.6, 18.2)	656	13.1 (9.2, 17.4)	0.04	13.3 (9.2, 18.0)*	14.2 (10.4, 18.2)	13.4 (9.4, 17.3)	12.7 (9.0, 17.4)
Subscapular skinfold, mm	898	21.6 (15.7, 28.6)	656	20.7 (14.8, 27.5)	0.04	21.2 (15.2, 28.5)*	22.6 (16.4, 28.7)	21.7 (15.2, 27.6)	20.0 (14.5, 27.3)
At visit 3^3^									
Weight, kg	957	52.7 (47.5, 59.1)	379	51.8 (46.9, 58.5)	0.24	52.4 (46.6, 59.1)	52.8 (48.0, 59.0)	52.5 (46.9, 59.4)	51.7 (47.0, 57.8)
Weight gain from recruitment, kg	957	7.2 ± 3.9	379	7.3 ± 3.9	0.74	7.4 ± 3.9*	6.9 ± 3.9	7.2 ± 3.6	7.3 ± 4.1
Triceps skinfold, mm	991	14.4 (10.6, 19.3)	399	13.3 (10.2, 18.2)	0.007	14.2 (10.3, 19.1)	14.7 (11.0, 19.4)	13.4 (10.1, 18.2)	13.3 (10.3, 18.1)
Triceps gain from recruitment, mm	991	0.5 ± 4.7	399	−0.1 ± 4.5	0.05	0.7 ± 4.4	0.3 ± 4.8	−0.5 ± 4.5	0.3 ± 4.6
Triceps gain from visit 1, mm	885	1.0 ± 3.6	321	0.3 ± 3.8	0.007	1.0 ± 3.4	0.9 ± 3.7	0.0 ± 3.6	0.6 ± 4.0
Subscapular skinfold, mm	991	23.4 (17.8, 29.4)	399	21.7 (17.0, 28.5)	0.03	22.9 (17.5, 28.5)	23.6 (18.4, 29.7)	21.7 (17.0, 29.4)	21.7 (17.1, 27.1)
Subscapular gain from recruitment, mm	991	1.2 ± 7.5	399	0.8 ± 7.2	0.43	1.4 ± 7.5	1.0 ± 7.4	1.0 ± 7.1	0.6 ± 7.2
Subscapular gain from visit 1, mm	885	1.6 ± 5.6	321	0.8 ± 5.3	0.03	1.5 ± 5.6	1.6 ± 5.5	0.9 ± 5.4	0.7 ± 5.2

1 Values are medians (IQRs), means ± SDs, or *n* (%). *Different from control, *P* < 0.05. OGTT, oral-glucose-tolerance test.

2 Median (IQR) gestation 10.1 (9.4, 12.0) wk.

3 Median (IQR) gestation 29.7 (29.3, 30.7) wk.

The prevalence of GDM (WHO 1999 criteria) (27) was 9.9%. Both in the intention-to-treat and per-protocol analyses, the prevalence was lower in the treatment group (intention-to-treat: 7.3% compared with 12.4%, *P*-difference = 0.008; OR: 0.56; 95% CI: 0.36, 0.86; and per-protocol: 7.5% compared with 13.1%, *P* = 0.01; OR: 0.54; 95% CI: 0.33, 0.86) (**Table 2**). This effect was independent of baseline and 28 wk skinfold measurements (**Table 3**) or baseline and 28-wk weight, or all of these measures combined.

**TABLE 2:** Prevalence of gestational diabetes and mean glucose and insulin concentrations according to allocation group^1^

	Treatment group		Control group		
	*n*	Value	*n*	Value	*P*
Intention-to-treat analysis (all women who became pregnant and attended for an OGTT)					
Gestational diabetes, WHO 1999 (27)	492	36 (7.3)	516	64 (12.4)	0.007
Gestational diabetes, WHO 2013 (29)	492	44 (8.9)	516	57 (11.1)	0.27
Diabetes in pregnancy, WHO 2013 (29)	492	6 (1.2)	516	3 (0.6)	0.33
Fasting glucose, mmol/L	492	4.21 (3.99, 4.55)	516	4.20 (3.95, 4.58)	0.40
Fasting insulin, IU/L	481	6.00 (4.00, 9.10)	508	6.05 (4.15, 9.10)	0.61
120-min glucose, mmol/L	484	5.66 (4.98, 6.54)	512	5.73 (5.01, 6.70)	0.14
Per-protocol analysis (subgroup of women who started supplementation ≥3 mo before conception)					
Gestational diabetes, WHO 1999 (27)	375	28 (7.5)	420	55 (13.1)	0.01
Gestational diabetes, WHO 2013 (29)	375	34 (9.1)	420	47 (11.2)	0.32
Diabetes in pregnancy, WHO 2013 (29)	375	4 (1.1)	420	3 (0.7)	0.71
Fasting glucose, mmol/L	375	4.19 (3.97, 4.51)	420	4.20 (3.95, 4.58)	0.97
Fasting insulin, IU/L	365	6.00 (4.00, 8.90)	412	6.00 (4.20, 9.20)	0.43
120-min glucose, mmol/L	370	5.64 (4.95, 6.59)	416	5.73 (5.01, 6.73)	0.16

1 Values are medians (IQRs) or *n* (%). OGTT, oral-glucose-tolerance test.

**TABLE 3:** Multiple logistic regression analysis for the effect of supplementation on GDM (intention-to-treat analysis)^1^

	Effect on GDM (normal, 0; GDM, 1), OR	95% CI	*P*
WHO 1999 criteria (27)			
Effect of intervention			
Control group	(ref)	(ref)	(ref)
Treatment group	0.6	0.4, 0.9	0.02
Maternal measurements			
Baseline subscapular skinfold, mm (logged)	1.9	1.0, 3.4	0.05
Subscapular gain from registration to visit 3, mm	1.0	1.0, 1.0	0.90
Height, cm	1.0	1.0, 1.1	0.75
Age, y (logged)	16.6	3.3, 83.2	0.001
Standard of Living Index, score (logged)	2.0	0.7, 5.4	0.19
Parity			
0	(ref)	(ref)	(ref)
1	1.4	0.8, 2.5	0.23
>1	1.1	0.5, 2.3	0.82
Compliance^2^			
Noncompliant	(ref)	(ref)	(ref)
Compliant	0.8	0.5, 1.3	0.39
Gestational age at visit 3, wk (logged)	0.9	0.0, 46.6	0.95
Intercept	0.0	0.0, 2.8	0.07
WHO 2013 criteria (29)			
Effect of intervention			
Control group	(ref)	(ref)	(ref)
Treatment group	0.8	0.5, 1.3	0.39
Maternal measurements			
Baseline subscapular skinfold, mm (logged)	1.5	0.8, 2.7	0.22
Subscapular gain from registration to visit 3, mm	1.0	1.0, 1.0	0.43
Height, cm	1.0	1.0, 1.1	0.27
Age, y (logged)	1.0	0.2, 4.9	0.97
Standard of Living Index, score (logged)	1.4	0.5, 3.6	0.50
Parity			
0	(ref)	(ref)	(ref)
1	1.5	0.9, 2.6	0.13
>1	1.0	0.5, 2.2	0.97
Compliance^2^			
Noncompliant	(ref)	(ref)	(ref)
Compliant	0.9	0.6, 1.4	0.53
Gestational age at visit 3, wk (logged)	1.9	0.0, 123.5	0.76
Intercept	0.0	0.0, 732.0	0.23

1 All variables shown were included in the model together, based on *n* = 837 pregnancies with complete data for all variables. GDM, gestational diabetes; ref, reference.

2 Categorical variable that was equal to 1 if the total number of supplements consumed in the 90 d before the last menstrual period date up to delivery divided by the total number it was possible to have eaten in that time was ≥0.5 (compliant); otherwise, 0 (noncompliant).

**FIGURE 1:**
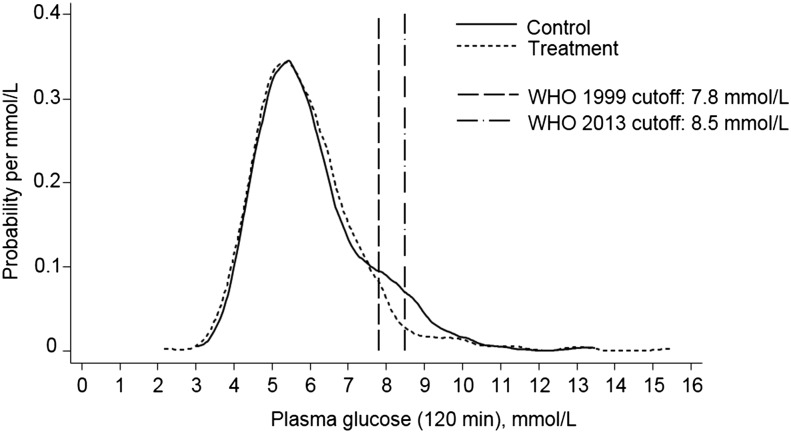
Kernel density plot of 120-min plasma glucose concentrations in the treatment and control groups (intention-to-treat analysis) and WHO cutoff values for gestational diabetes.

There was no difference between treatment and control groups when we used the WHO 2013 GDM criteria (29) (intention-to-treat: 8.9% compared with 11.1%, *P* = 0.27; OR: 0.79; 95% CI: 0.52, 1.20; and per-protocol: 9.1% compared with 11.2%, *P* = 0.32; OR: 0.79; 95% CI: 0.50, 1.26) or diabetes-in-pregnancy criteria (Table 2). Moreover, there were no significant differences between allocation groups in mean fasting or 120-min glucose concentrations, or fasting insulin concentration. A Kernel density analysis (**Figure 1**) explained these findings, as well as the discrepancy between 1999 (27) and 2013 (29) criteria. There were more control women than treatment women with 120-min glucose concentrations in the range 7.5–10 mmol/L (*P*-heterogeneity = 0.06 in frequencies in 3 glucose groups, including <7.5, 7.5–10.0, and >10.0). Frequencies of normal or very high glucose concentrations were similar in both allocation groups (Table 2)

There were no significant interactions between allocation group and maternal age, BMI, height, or parity in relation to any outcome.

Women who developed GDM were older and more adipose than women who did not (**Supplemental Table 4**, 1999 criteria). They had a similar prepregnancy intake of leafy green vegetables, fruit, and milk. Overall, 25% of women were vegetarian (ate no meat or fish); women who developed GDM ate nonvegetarian foods more frequently than women who did not develop GDM. One-third of women were anemic in early pregnancy and 17% were vitamin B-12 deficient, whereas only 1% were folate deficient; there were no differences in the prevalence of anemia or B-12 or folate deficiency between women who did and did not develop GDM. There were more preterm births in the GDM group (*P* = 0.002) and fewer small-for-gestational-age births, as well as more congenital anomalies and emergency Cesarean sections (all borderline significant ∼*P* = 0.1). Findings were similar for the 2013 criteria.

## Discussion

In a large randomized, controlled trial in women living in Mumbai slums, a daily micronutrient-rich snack starting preconceptionally and continuing throughout pregnancy almost halved the prevalence of GDM based on WHO 1999 criteria (27) (OR: 0.56; 95% CI: 0.36, 0.86). The effect was independent of maternal adiposity. There was no effect on GDM diagnosed based on WHO 2013 criteria (29).

Strengths of the trial include the individual random assignment, supervised supplementation, dating of pregnancies based on LMP and ultrasound, and standardized OGTTs. There are a number of important limitations to the GDM data. Only one-half the women chose to have an OGTT. This resulted in a fairly small sample size (100 cases of GDM) and could have biased the results. The main reason women did not attend for the OGTT was that blood testing is greatly disliked, and although many obstetricians were pleased to accept our OGTT results, others preferred to carry out their own, in which case women were understandably reluctant to have another OGTT with our research team. We did not use the results of other OGTTs, because of the variety of protocols and laboratories used. We do not have reliable data on history of GDM in earlier pregnancies; it is possible that women with a prior history of GDM were more likely to attend for an OGTT than those with no previous GDM history. We did not take blood 1 h after the glucose load, so it is possible that we missed some cases of GDM based on the 2013 diagnostic criteria (29). However, all these issues would be expected to affect both allocation groups equally, so we think such issues are unlikely to have created spurious or biased results. The prevalence of GDM in our study is well within the range expected for an urban Indian population (2, 17). Women who had an OGTT were older and of higher parity and socioeconomic status than were women who did not, and women in the treatment group were slightly lighter and less adipose preconceptionally than controls, but gained more weight during pregnancy. However, adjusting for these factors did not alter our findings. Because it was food-based, full blinding of our intervention was not possible. However, laboratory staff were blind to the women’s allocation, and it is difficult to see how lack of blinding could alter the women’s behavior in ways that would reduce GDM so markedly. Because of funding constraints, we had limited information on the women’s micronutrient status, which limits our ability to suggest mechanisms for the reduction in GDM. We measured only vitamin B-12 and folate, which have been linked to birth weight in Indian populations (32–34). We did, however, carry out a separate study in nonpregnant women in a similar slum community in Mumbai, using the same supplements, specifically to measure a range of micronutrients before and after 3 mo of supplementation (vitamin C, β-carotene, retinol, ferritin, folate, vitamin B-12, and homocysteine) and found that, of these, only β-carotene concentrations increased (35). It also would have been useful to record women’s physical activity to determine if this was part of the mechanism for the reduction in GDM. We have to consider the possibility that the intervention did not prevent GDM in the treatment group, but that the control snacks, which were lower in protein, increased the risk of GDM. This seems unlikely, because the control snacks contained less energy than the intervention snacks, and women in the control group did not gain more fat than those in the intervention group. Our results, in a predominantly vegetarian population with a very low baseline intake of leafy green vegetables, fruit, and milk, and in which the daily snack made a substantial difference in the intake of these foods, may not be generalizable to more affluent, nonvegetarian populations with a more diverse habitual diet. Neither was our trial designed to determine whether starting supplementation before conception rather than during pregnancy was important.

As far as we know, this is the first randomized trial in which it was possible to examine the effect of micronutrient-rich foods on GDM risk, albeit as a secondary outcome. The effect was large, translating to a number needed to treat of 20 (intention-to-treat analysis, 1999 criteria). Observational studies have shown that a higher ratio of polyunsaturated to saturated dietary fat, higher intake of carbohydrates relative to fat, and higher vitamin B-12, C, and D status are associated with a lower risk. The US Nurse’s Study found that higher “prudent diet” scores (a higher intake of fruit, leafy green vegetables, poultry, and fish) predicted a lower risk (RR in lowest intake quartile = 1.37; 95% CI: 1.09, 1.72) (36). There is similar prospective evidence of protection against T2DM (10–14, 37, 38). Two reviews of specific food groups found no relation between total fruit or vegetable intake and T2DM risk, but a subgroup of studies that gave separate information for leafy green vegetable intake showed an ∼14% lower risk of developing T2DM in the highest than in the lowest intake categories (13, 14).

We do not know which constituents of the snacks produced the effect. The main differences between the snacks were the fillings (leafy green vegetables, fruit, and milk in the intervention snacks compared with low-micronutrient vegetables in the control snacks). Other nutrients in the snacks came from the covering/binding ingredients and the cooking oil, which were similar in both groups, although the former were greater in quantity in the intervention snacks, resulting in 0.32 MJ more energy on average and 4 g more protein per snack. Leafy green vegetables contain the antioxidants β-carotene, vitamin C, and polyphenols. However, these have not prevented T2DM in randomized trials (39). Leafy green vegetables are rich in magnesium, a higher intake of which has been associated with a lower risk of T2DM (40) and which reduce fasting glucose in trials (41). The effect may be from FAs; leafy green vegetables are a rich source of long-chain ω-3 PUFAs, which may improve insulin sensitivity by influencing the properties of cell membranes (42). Leafy green vegetables also contain nitrates, which increase thermogenesis, oxygen consumption, and β-oxidation in rat adipocytes (43). The association between higher dairy intake and lower risk of future T2DM has been attributed to calcium, vitamin D, or whey protein (12). The snack format for this trial was chosen for pragmatic reasons after various preparations of the key foods were piloted. The fried snacks could be individually packaged, preventing contamination, and remained palatable after transportation to the supplementation centers. If leafy green vegetables, fruit, and milk, or any one of these foods, were the effective agents, we would not necessarily advocate their delivery in the form of a fried snack.

The different results from the 1999 (27) and 2013 (29) diagnostic criteria were explained by the fact that fewer women in the treatment group had 120-min glucose values in a middle impaired glucose tolerance range than controls (Figure 1). Glucose measurements below and above this range and mean glucose values were similar in both allocation groups. The WHO 1999 criteria defined GDM with the use of the same glucose cutoffs as for impaired glucose tolerance in the nonpregnant state (27). The WHO 2013 criteria were based on *1*) plasma glucose cutoff values associated with an OR of 1.75 (compared with mean values) for birth weight, newborn adiposity and cord blood C-peptide >90th percentile, and *2*) a simulation exercise suggesting that smaller numbers would need to be screened to prevent adverse outcomes (29). However, it is recognized that the cutoffs are still to some extent arbitrary (29, 44), because the adverse maternal and neonatal outcomes increase linearly across the range of glucose concentrations, with no apparent thresholds (3, 4). Our interpretation of our findings is that the supplement had no effect on glucose concentrations in women with normal glucose tolerance or in those with established diabetes in pregnancy, but that there was an intermediate group of women who were vulnerable to diabetes and whose metabolic competence was improved by the supplement. Our findings add to the debate about diagnostic cutoffs, and perhaps make a case for maintaining the impaired glucose tolerance range within the criteria for GDM.

We measured maternal vitamin B-12 and folate status because of data showing a high prevalence of vitamin B-12 deficiency in pregnant Indian women, especially in rural communities, that is thought to result from vegetarianism (45, 46), and an association between vitamin B-12 deficiency and GDM (9). There was no difference, however, in vitamin B-12 status in early pregnancy between the intervention and control groups, and no association between vitamin B-12 status and GDM in our study.

In conclusion, the results of this randomized, controlled trial suggest that improving women’s dietary micronutrient quality may have important protective effects against GDM. Because GDM was not the trial’s primary outcome and because of 50% nonparticipation for the OGTT, the findings would need to be replicated. However, they are consistent with observational research showing a lower risk of GDM and T2DM in association with the foods contained in the trial supplements.

## Footnotes

9 Abbreviations used: GDM, gestational diabetes mellitus; LMP, last menstrual period; OGTT, oral-glucose-tolerance test; T2DM, type 2 diabetes mellitus.
